# Do Men and Women Differ in Hematological Adaptations to 24 Weeks of Crossfit® Training?

**DOI:** 10.5114/jhk/170885

**Published:** 2023-10-11

**Authors:** Ana Cristina Barreto, Luis Leitão, Jeferson Vianna, Rodrigo Poderoso, Victor Machado Reis, Maria Cirilo-Sousa, Adenilson Junior, Marcelo Colonna, Gustavo Casimiro-Lopes, Jefferson Novaes

**Affiliations:** 1 Physical Education Department, Celso Lisboa University Center, Rio de Janeiro, Brazil.; 2 Sciences and Technology Department, Superior School of Education of Polytechnic Institute of Setubal, Setúbal, Portugal.; 3 Life Quality Research Centre (CIEQV), Leiria, Portugal.; 4 Physical Education and Sports Department, Federal University of Juiz de Fora, Juiz de Fora, Brazil.; 5 Physical Education Department, University of Unopar, Nilópolis, Brazil.; 6 Research Centre in Sports Sciences, Health Sciences and Human Development, CIDESD, Vila Real, Portugal.; 7 Physical Education Department, Federal University of Paraiba, João Pessoa, Brazil.; 8 Postgraduate Program of Physical Education, University of Cariri Regional, Crato, Brazil.; 9 Physical Education Department, Federal Techonology Institute of Paraiba, João Pessoa, Brazil.; 10 Physical Education Department, University Center of Augusto Motta of UNISUAM, Rio de Janeiro, Brazil.; 11 Physical Education Department, Estácio de Sá University, UNESA, Rio de Janeiro, Brazil.; 12 Physical Education and Sports Institute, Laboratory of Exercise Pathophysiology, Rio de Janeiro State University, Rio de Janeiro, Brazil.; 13 Physical Education and Sports Department, Federal University of Rio de Janeiro, Rio de Janeiro, Brazil.

**Keywords:** high intensity, interval training, red blood cells, white blood cells

## Abstract

Regular exercise can modulate the immune system functioning through changes in the number and function of leukocytes as well as in red blood cells and other typical blood markers. High intensity exercise promotes increases in cytotoxic activity, phagocytic capacity, chemotaxis and cell apoptosis. The aim of the study was to compare the chronic effects of a 24-week training program using CrossFit® methodology on hematological variables of men vs. women. Twenty-nine CrossFit® athletes (35.3 ± 10.4 years, 175.0 ± 9.2 cm, 79.5 ± 16.4 kg) participated in the study. The blood count, the lipid profile and glucose markers were measured every two months during the study period. The erythrocyte count and hemoglobin concentrations increased in months 4 and 6 in men and women, respectively. Hematocrit levels increased in men in months 2, 4 and 6, while in women only in month 6. Red cell distribution width increased in men in month 6 when compared to the value in month 2. Segmented neutrophils increased in men in month 6 and eosinophil levels increased in women in month 6. Differences between the two sexes were observed in monocytes levels at baseline, as well as in months 2, 4 and 6. Cross-Fit^®^ training increased red cell count indicators in both sexes, which may be related to increased erythropoiesis. Some white blood cell counts were altered and these differed between sexes. The number of lymphocytes remained stable throughout the experiment.

## Introduction

Exercise combined with scientific advances allowed the improvement of different exercise training methodologies in the past years, updating and innovating the field of sports training and fitness. A training methodology increasingly used to improve sports performance and physical condition is CrossFit®, a physical training program characterized by constantly varied functional movements executed at relatively high intensity through gymnastics, metabolic conditioning and weightlifting (Kraemer et al., 2016; Leitão et al., 2021; Montalvo et al., 2017). The exercises used are combined and performed in successive repetitions with limited recovery time or no rest in between at all (Leitão et al., 2021; Weisenthal et al., 2014). This training methodology promotes cardiorespiratory and strength adaptations, including increased maximal VO_2_ and muscle strength (Leitão et al., 2021). Studies on CrossFit® have been preferably focused on biological measures able to predict performance (Gómez-Landero et al., 2020) and performance analysis during competitions (Mangine et al., 2021).

The high intensity used in CrossFit® generates significant changes in physiological, metabolic, inflammatory, hormonal, hemodynamic, autonomic and biochemical responses (Faelli et al., 2020; Jacob et al., 2020). Regular exercise can modulate functioning of the immune system through changes in the number and function of leukocytes, and the magnitude and direction of these changes are related to the volume, the intensity and the type of exercise (Krinski et al., 2010; Żak and Pokora, 2017). In turn, intense physical exercise promotes leukocytosis during and immediately after exercise (Sand et al., 2013; Suzuki et al., 2020) leading to an increase in cytotoxic activity, phagocytic capacity, chemotaxis and cell apoptosis. Gomes et al. (2020) showed a significant increase in the amount of white blood cells, neutrophils, lymphocytes and monocytes after a CrossFit® protocol, and individuals with longer experience had a higher number of lymphocytes when compared to beginners, a fact that may be related to a greater ability to recover between training sessions due to the immune system's memory to this type of stress. Poderoso et al. (2019) demonstrated a reduction in the CD8 T lymphocyte count in the first four months of training, although the CD4 cells were not altered, suggesting an immune system adaptation to chronic training. Petersen and Pedersen (2005) along with Bachero-Mena et al. (2017) supported that physically active subjects exhibited reduced inflammatory responses due to the improvement in immune status as a chronic response to physical exercise. However, Żak and Pokora (2017) and Fortunato et al. (2018) observed that trained individuals presented a lower number of white cells when compared to sedentary ones, which contributes to the susceptibility to infections.

Previous research has analyzed adaptations to 14-week CrossFit® training (Choi et al., 2017), but prolonged adaptations (i.e., 24-week training) have not been examined before. An exception is a study by Poderoso et al. (2019), yet hematological adaptations were assessed for white blood cells only.

There are several benefits that occur with exercise in the immune system and some hematological variables may also change over time. However due to being a recent exercise program, to date, no studies are reported on white blood cell and red blood cell adaptations to chronic CrossFit® training. Therefore, the aim of the present study was to compare the chronic effects of a 24-week training program using CrossFit® methodology on hematological variables of men and women.

## Methods

### Participants

Twenty-nine volunteers involved in Cross-Fit^®^ were included in this study. Both men (n = 17; 34.7 ± 7.6 years, 89.2 ± 7.2 kg, 180.8 ± 4.8 cm, 27.3 ± 2.2 kg/m^2^, 17.6 ± 2.1 BF%, 9.4 ± 2.7 years of training experience) and women (n = 12, 36.2 ± 13.7 years, 65.6 ± 15.7 kg, 166.8 ± 7.5 cm, 23.6 ± 5.7 kg/m^2^, 23.5 ± 4.8 BF%, 8.8 ± 1.9 years of training experience) were recruited from a CrossFit® affiliate to participate in the study. The inclusion criteria were: minimum training experience of six months; no history of muscle or joint injury; not taking any drug or nutritional supplement nor alcohol; and a minimum program attendance of 85%. Study exclusion criteria were: presence of any injury or osteoarticular problems that could restrict any exercise; heart problems or medical contraindications (e.g., surgeries); practice of any other exercise program in the last month; and reporting to use drugs, nutritional supplements or alcohol throughout the experiment. The study was approved by the local ethics committee (Doc46-CE-UTAD-2020) and all volunteers signed an informed consent form in accordance with the Helsinki Declaration (1964) and the Nuremberg Code (1947) concerning research involving human subjects.

### Measures

The 24-week CrossFit^®^ training protocol was performed five days a week, followed by two recovery days (weekend), for about 60 minutes per session. The sessions were constantly varied although they maintained the combination of metabolic conditioning, weightlifting and calisthenics. All sessions were supervised by CrossFit® certified coaches. The program sessions followed the same structure: joint mobility, a warm-up, a technical part, the workout of the day (WOD) and a cool-down period. All the 120 sessions were performed at a CrossFit^®^Inc affiliated center and supervised by a Level 1 certified CrossFit^®^ professional. Males and females performed the same training sessions with females lifting 30% lower loads in weightlifting. The volunteers were instructed to avoid any other type of physical exercise and to maintain their daily living and eating habits.

Body mass (kg), body fat content (%BF, %) and body mass index (BMI, kg·m^−2^) were assessed through a bioelectrical impedance analysis (OMRON BF 303, Matsusaka, Japan), and body height was measured with a stadiometer (Seca, Hamburg, Germany).

The red blood cell (RBCs) (erythrocytes, hemoglobin, hematocrit, mean corpuscular volume (MCV), mean corpuscular hemoglobin (MCH), mean corpuscular hemoglobin concentration (MCHC), red cell distribution width (RDW)) and white blood cell (WBCs) (leukocytes, monocytes, segmented neutrophils, lymphocyte, eosynophils and basophils) counts and the lipid profile (triglycerides, HDL, LDL, cholesterol and glycemia) values were determined through blood samples collected twice at each measurement point (T0, T2, T4, T6), once in the morning to control for any variations due to the hormonal circadian rhythm, and 12 h after training. For each sample, 5 mL of blood was drawn from the antecubital vein by a qualified professional and sent to a specialized medical laboratory for analysis. The samples were transferred into an EDTA treated Vacutainer® tube and carried in an isolated box, and the serum was isolated and stored at −4⁰C until the analysis.

### Design and Procedures

This was a six-month study consisting of 24 weeks of CrossFit^®^ training with responses being verified every two months of training. Blood samples were taken at the beginning of the training program (T0) and every two months throughout the training period (T2, T4, T6). All assessments were conducted in the same location and by one experienced researcher. Participants reported to blood testing in a fasting state with a minimum recovery of 48-h post exercise.

### Statistical Analysis

Data normality was checked with the Shapiro-Wilk test and comparisons between male and female athletes were performed using an unpaired Student’s *t*-test. When unequal variances were detected by the Levene’s test, we used the Welch test instead. To compare between both sexes all variables over time, we used repeated measures ANOVA (2 x 4) followed by the Bonferroni post-hoc test when necessary. Omega squared (ω^2^) was adopted to determine the magnitude of variance for effect with the following thresholds: no effect (<0.010); small (0.010 to 0.059); moderate (0.06 to 0.139) and large (≥ 0.14) effect. According to ε values Greenhouse-Geisser or Huynh-Feldt correction was applied in the event of sphericity violation detected by the Mauchly’s test. Statistical significance was set at *p* < 0.05. Additionally, we also used the Bayes factor bound (BFB) as an alternative measure of evidence considering the odds in favor of the alternative hypothesis relative to the null hypothesis related to the observed data. The size calculation of our sample was performed with G*Power (ver. 3.1.9.7; Heinrich-Heine-Universität Düsseldorf, Düsseldorf, Germany) with an *N* of 12 individuals in each group for a power of 0.8, α = 0.05, correlation coefficient of 0.5, nonsphericity correction of 1, and an effect size of 0.4. All data analyses were performed with Jasp Software (Jasp version 0.14.1, Amsterdam, Netherlands).

## Results

The training program promoted significant increases in months 4 and 6 in men (∆ = +0.2, *p* < 0.001) and women (∆ = +0.3, *p* < 0.001) in the erythrocyte count, both with large effects (F_2,47_ = 55.367 *, p* < 0.001, ω^2^ = 0.148; BFB = 5.3x10^3^). Hemoglobin concentrations changed significantly with exercise showing a moderate effect (F_2.46_ = 1176, *p* < 0.001, ω^2^ = 0.078; BFB = 4.0 x 10^3^), increases in months 4 and 6 in men (∆ = +0.5, *p* < 0.001 and ∆ = +0.7, *p* < 0.001) and women (∆ = +0.7, *p* < 0.001 and ∆ = +0.9, *p* < 0.001), respectively. Hematocrit levels also changed with a large effect (F_2.47_ = 55.367, *p* < 0.001, ω^2^ = 0.148; BFB = 5.6 x 10^3^), being increased in men after 2 months (∆ = +1.2, *p* = 0.001), 4 months (∆ = +1.6, *p* < 0.001) and 6 months (∆ = +1.8, *p* < 0.001) of training, while in women only they increased after 6 months of training (∆ = +1.4, *p* = 0.012). The RDW showed a small change as a result of exercise (F_2.54_ = 5.522, *p* = 0.007, ω_2_ = 0.047; BFB = 13.2) and interaction between the factors “time” and “sex” (F_2.54_ = 3.987, *p* = 0.021, ω^2^ = 0.032; BFB = 4.5).

Sex differences were observed in erythrocytes (F_1.27_ = 36.926, *p* < 0.001, ω^2^ = 0.391; BFB = 16.0 x 10^3^), hemoglobin (F_1.27_ = 30.384, *p* < 0.001, ω^2^ = 0.344; BFB = 4.0 x 10^3^) and hematocrit (F_1.27_ = 37.501, *p* < 0.001, ω^2^ = 0.395; BFB = 17.9 x 10^3^) at all time points with a large effect, while for MCV moderate effects were observed (F_1.27_ = 4.760, *p* = 0.038, ω^2^ = 0.063; BFB = 2.4). On the other hand, the concentrations of hemoglobin, hematocrit, erythrocytes, MCV, MCH and MCHC did not changed in response to exercise neither between the factors “time” nor “sex” (Table 1).

**Table 1:** Red Blood Cell counts in both men and women.

Variable	Sex	Baseline values	Month 2	Month 4	Month 6
Erythrocyte (10^12^/ L)	F	4.2 ± 0.4	4.3 ± 0.3	4.5 ± 0.3*	4.6 ± 0.4*
	M	4.9 ± 0.3^#^	5.0 ±0.2^#^	5.1 ± 0.3*^#^	5.2 ± 0.3*^#^
Hemoglobin (g/ dL)	F	12.9 ± 1.2	13.2 ± 1.2	13.6 ±1.0*	13.8 ± 1.1*
	M	15.0 ± 1.0^#^	15.3 ± 0.9^#^	15.5 ± 0.9*^#^	15.7 ± 0.8*^#^
Hematocrit (%)	F	38.4 ± 2.9	39.0 ± 2.7	39.6 ± 2.7	39.8 ± 2.8*
	M	43.6 ± 2.6^#^	44.7 ± 2.2^#^	45.2 ± 2.2*^#^	45.4 ± 2.1*^#^
MCV (µm^3^)	F	90.4 ± 3.7	90.7 ± 4.1	90.9 ± 4.0	90.7 ± 4.1
	M	87.7 ± 2.3	88.3 ± 1.9	88.4 ± 2.1	88.7 ± 2.1
MCH (pg/ cell)	F	30.7 ± 1.4	30.8 ± 1.2	30.9 ± 1.1	30.9 ± 1.2
	M	30.1 ± 1.0	30.1 ± 1.1	30.6 ± 1.5	30.4 ± 1.1
MCHC (Hb/ cell)	F	33.7 ± 1.1	33.4 ± 1.0	33.9 ± 1.2	34.1 ± 1.1
	M	34.3 ± 1.2	34.0 ± 1.3	34.3 ± 1.2	34.7 ± 1.1
RDW (%)	F	12.9 ± 0.5	13.1 ± 0.5	13.1 ± 0.4	13.2 ± 0.4
	M	12.9 ± 0.4	12.8 ± 0.4	12.9 ± 0.4	13.2 ± 0.3^$^

Values are means (± standard deviations). Mean corpuscular volume (MCV); mean corpuscular hemoglobin (MCH); mean corpuscular hemoglobin concentration (MCHC); red cell distribution width (RDW). * Indicates a difference from the baseline values (*p* < 0.05); # indicates a difference between males and females at the same time point (*p*< 0.05); $ indicates a difference from the measure in month 2 (*p* < 0.05).

Considering monocyte levels, differences between sexes were observed with a large effect (F_1.27_ = 20.072, *p* < 0.001, ω^2^ = 0.254; BFB = 332.1), at baseline (∆ = +204.0, *p* = 0.01), at months 2 (∆ = +215.1, *p* = 0.007), 4 (∆ = +212.8, *p* = 0.007) and 6 (∆=+214.5, *p* = 0.007). Leukocytes showed only a difference with a moderate effect between the sexes (F_1.27_ = 4.838, *p* = 0.037, ω^2^ = 0.064; BFB = 3.0), however, without noticing differences in any specific period of time. No interactions between the factors “time” and “sex” were observed in the levels of leukocytes, lymphocytes, eosinophils, monocytes and basophils. Likewise, there were no sex differences for lymphocytes, eosinophils and basophils (Table 2).

**Table 2:** White Blood Cell counts in men and women.

Variable	Sex	Baseline values	Month 2	Month 4	Month 6
Leukocytes (10^3^/ mm^3^)	F	5820.0 ± 2297.9	5470 ± 909.6	5846.2 ± 826.2	5987.1 ± 1,153.4
	M	6652.9 ± 1353.9	6697.0 ± 1,448.2	6261.2 ± 1,799.9	7175.3 ± 1,444.5
Monocytes (10^3^/ mm^3^)	F	378.1 ± 128.4	400.9 ± 142.6	431.8 ± 107.0	476.7 ± 109.6
	M	582.1 ± 161.5#	616.0 ± 135.3#	644.7 ± 189.8#	691.2 ± 153.6*#
Segmented (10^3^/ mm^3^)	F	2320.8 ± 708.7	2402.4 ± 668.2	2489.2 ± 684.1	2565.0 ± 694.3
	M	3198.6 ± 934.2	3198.8 ± 1,035.5	3557.0 ± 1,172.9	3701.7 ± 1,175.2*
Lymphocytes (10^3^/ mm^3^)	F	2206.1 ± 408.8	2,131.2 ± 403.2	2302.6 ± 396.6	2425.8 ± 427.4
	M	2536.7 ± 553.9	2458.2 ± 501.8	2503.5 ± 520.3	2644.7 ± 492.2
Eosynophils (10^3^/ mm^3^)	F	203.2 ± 153.6	265.0 ± 224.2	249.0 ± 189.4	278.7 ± 216.3*
	M	191.5 ± 102.7	190.6 ± 110.2	190.0 ± 109.5	203.5 ± 153.7
Basophils (10^3^/ mm^3^)	F	40.5 ± 19.3	38.5 ± 13.7	36.2 ± 12.3	36.7 ± 11.7
	M	38.3 ± 17.0	38.8 ± 14.5	36.2 ± 11.9	39.4 ± 9.6

Values are means (± standard deviations). * Indicates a difference from the baseline values (*p* < 0.05); # indicates a difference between males and females at the same time point (*p*< 0.05).

The training program promoted small changes in cholesterol levels (F_2.57_ = 4.222, *p* = 0.017, ω^2^ = 0.014; BFB = 5.2) showing a decrease in men at month 6 compared to month 2 (∆ = −21.2, *p* = 0.016). There were also small changes in HDL levels (F_2.68_ = 3.794, *p* = 0.019, ω^2^ = 0.018; BFB = 4.8), with significant increases observed in women at month 2 (∆ = +7.0, *p* = 0.022). Besides, significant differences were also observed between sexes, where women showed higher values of HDL in all time points (F_1,27_ = 23.626, *p* < 0.001, ω^2^ = 0.288; BFB = 828). Triglyceride levels did not show significant effects of the factor “time” or the interaction “time” and “sex”, but showed moderate differences between both sexes (F_1.27_ = 4.330, *p* = 0.047, ω^2^ = 0.056). No changes were observed for LDL and blood glucose in any of the conditions evaluated (Table 3).

**Table 3:** Lipid profile and glycemia in men and women.

Variable	Sex	Baseline values	Month 2	Month 4	Month 6
LDL (mg/ dL)	F	103.5 ± 29.5	107.4 ± 23.3	103.7 ± 25.8	102.3 ± 24.6
	M	118.3 ± 3 9.3	132.6 ± 51.7	119.7 ± 32.7	119.7 ± 34.2
HDL (mg/ dL)	F	56.4 ± 11.5	63.4 ± 17.1	61.1 ± 13.8	62.7 ± 12.6
	M	42.4 ± 5.9	42.6 ± 9.4*	42.8 ± 8.4	44.9 ± 6.3
Cholesterol (mg/ dL)	F	177.4 ± 37.1	184.2 ± 37.9	178.1 ± 37.7	175.1 ± 38.4
	M	187.1 ± 43.8	205.3 ± 55.2	188.5 ± 41.4	184.1 ± 38.6^$^
Triglycerides (mg/ dL)	F	91.5 ± 30.8	84.2 ± 25.9	80.7 ± 21.8	79.2 ± 18.7
	M	123.3 ± 62.0	124.5 ± 70.2	124.6 ± 62.6	110.8 ± 47.9
Glycemia (mg/ dL)	F	86.3 ± 6.3	87.2 ± 5.7	86.7 ± 5.2	86.0 ± 4.3
	M	87.5 ± 5.8	87.9 ± 4.3	87.0 ± 5.8	87.4 ± 3.7

Values are means (± standard deviations). Low density lipoprotein (LDL); high density lipoprotein (HDL). * Indicates a difference from the baseline values (*p* < 0.05); $ indicates a difference from the measure in month 2 (*p* < 0.05).

## Discussion

The aim of the study was to compare the chronic effects of a 24-week training program using CrossFit® methodology on hematological variables of men and women The main results showed that a six-month CrossFit^®^ training program resulted in: a) no significant changes in the hematology indexes, lipid profile and glycemia; b) an increase in the count of erythrocytes, hemoglobin concentration and hematocrit in both men and women; and c) the immune system variables increased in specific components such as monocytes, segmented neutrophils and eosinophils, which varied according to sex.

Data from the present study did not show significant differences in the lipid profile and glucose levels after the training program, neither in males nor females. Choi et al. (2017) also examined the effects of a CrossFit® training program on body composition and the blood profile in a group of 22 Korean students, and observed that 14 weeks of training were effective in changing the body composition. However, those authors found no significant changes in the lipid profile. Hence our data agree with these of Choi et al. (2017).

Bacharo-Mena et al. (2017) analyzed hematological variables during a complete 800-m running athlete's season and observed a reduction in hematocrit, mean corpuscular volume (MCV), mean corpuscular hemoglobin content (MCHC) and the amount of white blood cells and monocytes. Bussollaro et al. (2018) observed hematological reductions in MCV, mean corpuscular hemoglobin (MHC), MCHC and an increase in eosinophils over a year in professional soccer players. Allis et al. (2015) reported that running on a treadmill at high intensity resulted in an increase in hemoglobin, hematocrit and RBC when compared to baseline values due to hemoconcentration induced by exercise and, consequently, through the plasma volume variation. Montero et al. (2017) showed that the increase in red blood cells volume was preceded by the increase in the erythropoietin concentration, which in turn occurred due to the reduction in the hematocrit. This reduction is due to the expansion of the plasmatic volume resulting from the transient activation of the renin-angiotensin-aldosterone system which increases blood protein circulation and baroreflex action in response to increased central venous pressure. Lippi et al. (2014) found that moderate-intensity exercises induced a variation in the volume and size of red blood cells (RDW). The number of RBCs and hemoglobin were significantly reduced after exercise, while the mean values of variation in RDW increased after training.

Physical exercise has been associated with an increase in young RBC turnover which results in a steady state of erythrocyte; this allows more efficient oxygen release by hemoglobin and greater activity of the 2,3-diphosphoglycerate enzyme (2,3 DPG). The increase in erythropoietin (EPO) promotes an incorporation of iron into red cells, which is reflected in the greater capacity to transport oxygen, being regulated by androgenic hormones such as testosterone, which, in part, explains the greater mass of red cells in men when compared to women (Tak et al., 2013; Tibana et al., 2018). Thus, although no significant differences were found in all the red cell variables, it can be suggested that CrossFit^®^ could have generated chronic adaptations in the neurohumoral activation mechanisms. This adaptation based on changes in the variables of erythrocytes, hemoglobin and hematocrit enabled changes in the capacity to transport and consume oxygen, and in exercise tolerance.

In the immune system cells, we observed differences in response to training that varied according to sex. Monocyte levels had higher values in men at all time intervals that were evaluated, and after the training period they were increased in the same group. Similarly, the levels of segmented neutrophils also showed an increase in their count after 24 weeks of CrossFit^®^ training. On the other hand, women showed an increase in eosinophils in response to training after the same period of time. Node et al. (2010) observed a significant reduction in the number of leukocytes, monocytes, and neutrophils in a sample of overweight women undergoing a six-week aerobic exercise program of moderate intensity and short duration. Those authors suggested that a reduction in the amount of neutrophil and monocyte cells might be associated with a reduction in triglycerides and the body mass index (BMI) and an increase in insulin sensitivity and oxygen consumption. Koopman et al. (1981) stated that in young women, monocytes had lower cytosolic activity when compared to men. The release of lysosomal enzymes implies the cytosolic activity of human monocytes and would be related to the release of sex hormones, which would explain the lower activity found in women. In the present study, we observed a significant increase in the number of monocytes after six months of training only in the group of men, which could be related to this particular type of training.

Physical exercise activates immunocompetent cells through changes in the concentration of estrogen, concentration of growth hormone and prolactin that stimulate the release of cytokines, i.e., proteins that are inhibitors of the hypothalamic-gonad axis. After high intensity exercise, transient immunosuppression occurs in the number of monocytes, which could explain the lack of elevation of these cells in women (Blanks et al., 2020; Faelli et al., 2020). The increase in the activity of the sympathetic nervous system and, consequently, the release of catecholamines during exercise promotes post-exercise monocyte recruitment, and this response is attenuated as chronic adaptations accumulate (Suzuki et al., 2020). Physical exercise delays the aging of monocytes, changing them towards a less inflammatory phenotype and affecting their morphology, which favors an increase in the area of monocytes; this enhances phagocytic activity (Blanks et al., 2020). These mechanisms may also justify the findings related to monocytes after six months of CrossFit^®^ training. Poderoso et al. (2019) observed that CrossFit^®^ training showed a significant effect in reducing cortisol levels in men and in CD8 T lymphocytes of both sexes, while the levels of CD4 did not show significant changes, suggesting an adaptation of the immune system to training.

The differentiated levels of testosterone between both sexes are consistent with the natural dimorphism presented by this hormone and appear to have an important effect on the immune system (Petersen et al., 2005). Heavens et al. (2014) showed that the CrossFit^®^ program increased the number of androgen receptors and endogenous testosterone. Markmann et al. (2020) indicated that signaling androgen played an important role in the inactive and adaptive processes of the immune system, including the development of T and B lymphocyte cells, the production, maturation and function of neutrophils, in addition to the activation of monocytes and macrophages. Testosterone signaling action, via androgen receptors, demonstrates that neutrophils expressing androgen receptors are strongly impacted by hormonal changes and could explain the differentiated effects between sexes on the tissue regeneration mechanism in response to the training program.

McKune et al. (2004) observed an increase in the non-allergic activation of blood eosinophils immediately after the ultramarathon and it remained high until 72 hours later when compared to pre-exercise values in a sample composed of 11 experienced individuals (6 men and 5 women; 43.0 ± 9.8 years). Those authors suggested that strenuous exercise would cause bronchial hyperactivity that increases fluid loss and, subsequently, generates an osmotic change in the bronchial epithelium. This process attracts eosinophils to the airway mucosa through mechanisms of action related to interleukin 5 (IL-5) and tumor necrosis factor alpha (TNF-α). In the present study, the results showed that the number of eosinophils increased significantly after six months of training when compared to values prior to the exercise program, and this was observed only in women. On the other hand, when submitted to a high-intensity, short-duration exercise protocol, women demonstrate a faster inflammatory response and more muscle damage when compared with men, a fact that may be related with greater non-allergic activation of eosinophils in women, as means to help in the removal and cell repair process of non-phagocytable fragments (Heavens et al., 2014).

There are some aspects of the study that may limit the generalization of the results herein. The dietary routine of participants was not controlled for and we did not include a non-exercising control group.

In conclusion, the red cell indicators related to erythropoiesis increased following the CrossFit^®^ training program in both men and women. Contrarily, the immune system measures along the study seemed to vary between both sexes possibly due to the concentrations of sex hormones, although the lymphocyte count remained stable throughout the exercise program.
